# Electrofluidic control of bioactive molecule delivery into soft tissue models based on gelatin methacryloyl hydrogels using threads and surgical sutures

**DOI:** 10.1038/s41598-020-63785-z

**Published:** 2020-04-28

**Authors:** Joan M. Cabot, Luciana Y. Daikuara, Zhilian Yue, Patricia Hayes, Xiao Liu, Gordon G. Wallace, Brett Paull

**Affiliations:** 1 Australian Centre for Research on Separation Science (ACROSS) and ARC Centre of Excellence for Electromaterials Science (ACES), School of Natural Sciences, Faculty of Chemistry, University of Tasmania, Tasmania, TAS 7005 Australia; 2 ARC Centre of Excellence for Electromaterials Science (ACES), Intelligent Polymer Research Institute (IPRI), University of Wollongong, Wollongong, NSW 2522 Australia

**Keywords:** Biological techniques, Microfluidics

## Abstract

The delivery of bioactive molecules (drugs) with control over spatial distribution remains a challenge. Herein, we demonstrate for the first time an electrofluidic approach to controlled delivery into soft tissue models based on gelatin methacryloyl (GelMA) hydrogels. This was achieved using a surgical suture, whereby transport of bioactive molecules, including drugs and proteins, was controlled by imposition of an electric field. Commonly employed surgical sutures or acrylic threads were integrated through the hydrogels to facilitate the directed introduction of bioactive species. The platform consisted of two reservoirs into which the ends of the thread were immersed. The anode and cathode were placed separately into each reservoir. The thread was taken from one reservoir to the other through the gel. When current was applied, biomolecules loaded onto the thread were directed into the gel. Under the same conditions, the rate of movement of the biomolecules along GelMA was dependent on the magnitude of the current. Using 5% GelMA and a current of 100 µA, 2 uL of fluorescein travelled through the hydrogel at a constant velocity of 7.17 ± 0.50 um/s and took less than 8 minutes to exit on the thread. Small molecules such as riboflavin migrated faster (5.99 ± 0.40 μm/s) than larger molecules such as dextran (2.26 ± 0.55 μm/s with 4 kDa) or BSA (0.33 ± 0.07 μm/s with 66.5 kDa). A number of commercial surgical sutures were tested and found to accommodate the controlled movement of biomolecules. Polyester, polyglactin 910, glycolide/lactide copolymer and polyglycolic acid braided sutures created adequate fluid connection between the electrodes and the hydrogel. With a view to application in skin inflammatory diseases and wound treatment, wound healing, slow and controlled delivery of dexamethasone 21-phosphate disodium salt (DSP), an anti-inflammatory prodrug, was achieved using medical surgicryl PGA absorbable suture. After 2 hours of electrical stimulation, still 81.1% of the drug loaded was encapsulated within the hydrogel.

## Introduction

In 1912, Zorab^1^ treated glaucoma by draining the anterior chamber to the subconjunctival space with a single silk thread^2,3^. Silk acted as a wick, removing fluid to reduce the intraocular pressure. After that, various types of drainage approaches were proposed, including the use of platinum^4^ thread/wire, acrylic^5^, and polydimehylmethacrylate microtubules^6^, and even more complex methods attempting to control the pressure using pumps^7^. Thread and textiles have recently gained considerable attention as low-cost substrates for microfluidics and biosensor applications, based upon their mechanical strength and the ability to facilitate and direct fluid movement. Fluid flow in threads arise from wicking processes as a result of capillary forces generated within the gaps between directionally aligned fibres. Threads have been used in this way within a variety of applications, including bioanalysis^8–14^, and embedded in a hydrogel as a network for chemotaxis studies^15^ or multilayer feeding of cell cultures^16^. This capillary action subsequently drives the fluid along the thread, and it is directly related to the material hydrophilicity on the surface. For instance, the presence of waxes such as those composed of long fatty acid chains found on some natural fibres like cotton as well as contamination on synthetic fibres such as polyester have been observed to interfere with wicking in threads^13,14^.

Hydrogels can be produced to simulate the characteristics of native extracellular matrices and provide a 3D support for cellular growth and tissue formation^17,18^. The ability of hydrogels to mimic the mechanical properties of soft tissue has seen them extensively applied in biomedical applications^19^, particularly presenting as an attractive substrate for biomedical implants^20,21^ and tissue engineering^18,22^. Among the natural polymers, gelatin methacryloyl (GelMA) presents several interesting advantages, as it is in fact denatured collagen and is a photopolymerizable hydrogel^23–27^. The mechanical properties of a variety of soft tissues, including neural tissue, vascularization, cartilage, bone, skin, skeletal muscle, cardio, liver, and kidney can be reproduced through use of appropriate GelMA concentrations and the photo-crosslinking conditions^27–31^. Here we use GelMA based hydrogels as tissue models to demonstrate an effective way to control the delivery and spatial distribution of biomolecules.

Threads can be readily integrated into the gel and they provide for an easy and robust way to transport liquid in or out of a gel. Direct application of biomolecules onto a thread or yarn is trivial and enables dispatch at a targeted location^9^. Herein, we demonstrated the use of electrofluidics – precise control and manipulation of fluids at the microscale by the use of an external electric field – on threads to deliver biomolecules into and throughout a gel, via control of an applied electric field, to provide a simple approach involving no moving parts or pumps.

## Materials and Methods

### Materials and reagents

Gelatin (porcine skin, Type A, ~300 g Bloom), methacrylic anhydride, dexamethasone 21-phosphate disodium salt (DSP), lithium phenyl-2,4,6-trimethylbenzoylphosphinate (LAP), 4-(2-hydroxyethyl)piperazine-1-ethanesulfonic acid (HEPES), fluorescein, riboflavin, rhodamine B, fluorescein isothiocyanate labelled bovine serum albumin (FITC-BSA) and fluorescein isothiocyanate labelled dextran (FITC-dextran) were purchased from Sigma-Aldrich (Australia). Phosphate buffered saline (PBS) (Medicago, Sweden), tris(hydroxymethyl)aminomethane (TRIS) (Amresco, US), acetonitrile (Chem-Supply Pty Ltd, Australia), ammonium acetate (Ajax Finechem Pty Ltd, Australia) were also used. Solutions were prepared in Milli-Q water with a resistivity of 18.2 MΩ cm. Acrylic (diameter (Ø) 671 ± 58 µm, 4 ply, Marvel Soft Baby, Bella Baby, Turkey) threads were evaluated for delivery studies. Nine different surgical sutures for general soft tissue approximation and/or ligation were employed for drug delivery studies: polyester surgical suture (USP 4/0 KeeboMed Inc, US); lactomer surgical suture (USP 3/0 Polysorb, Covidien, Ireland); poliglecaprone 25 surgical suture (USP 3/0 Monocryl), polypropylene surgical suture (USP 3/0 Prolene), nylon surgical suture (USP 3/0 Ethilon), silk surgical suture (USP 3/0) and polyglactin 910 surgical suture (USP 3/0 Vicryl) obtained from Ethicon (Kansas City, MO, US); polyamide surgical suture (USP 0 Supramid) and polyglycolic acid surgical suture (USP 0 Surgicryl PGA) supplied by SMI (Hünningen, Belgium). All these surgical sutures meet the criteria of ISO 9001 and ISO 13485, which describe a quality management system for development, manufacturing and sales of medical products and employ them for the main medical indications.

### Synthesis of gelatin methacryloyl (GelMA)

GelMA was synthesized by a direct reaction of gelatin with methacrylic anhydride. Gelatin was dissolved in PBS (10% w/v) at 50 °C for 1 hour. The solution was autoclaved and then stirred at 50 °C, while methacrylic anhydride (0.6 mL/g gelatin) was added drop-wise over a period of approximately 3 minutes. The reaction was continued for 4 hours and terminated by adding 300 mL of PBS. Sodium hydroxide was added drop-wise to adjust the pH (pH = 7) using a WP-80 Handheld pH/mV meter. Then, the solution was dialyzed against deionized water at 40 °C for 3 days (cellulose membrane, molecular weight cut-off: ~12 kDa). The above procedure was conducted under a sterile condition in a biosafety cabinet. Lastly, the dialyzed GelMA solution was lyophilized and stored at −20 °C until further use.

### Preparation of GelMA hydrogels

GelMA hydrogels were prepared by dissolving various amounts of GelMA (2.5–7.5%) in two buffer solutions, PBS (5.0%) and TRIS/HEPES (5 mM), respectively. For each solution, LAP was then added to the solution with a final concentration of 0.06%, loaded into a customised hydrogel chamber and crosslinked via UV light (400 nm) exposure for 45 seconds, unless specified otherwise. GelMA volume was kept constant at 15 mm^3^ (2.5 × 2.5 × 2.4 mm, length × width ×height).

### Mechanical properties of GelMA hydrogels

The mechanical properties of hydrogels were measured by indentation (EZ-S mechanical tester, Shimadzu) using a compression mode. Each sample was prepared by casting a polymer solution (15 μl) in a 2.5 × 2.5 × 2.5 mm custom mold. A cylindrical flat-ended stainless-steel indenter (990 μm in diameter) was attached to a 10 N load cell and programmed to apply force to the hydrogel surface at a controlled displacement rate of 0.5 mm/min. The Young’s modulus of the hydrogel was calculated through the slope of the applied force against indentation depth (0–100 μm) plot. The radius of the indenter tip was 0.495 mm. The slope of the linear fit was then used to calculate the hydrogel modulus (E). Young’s modulus was calculated for 2 different GelMA concentration (5.0, 7.5%) and 3 cross-linking times (30, 45, and 60 s). 5 replicates were carried for each determination.

### Nuclear Magnetic Resonance (NMR)

NMR was performed to verify the degree of functionalization of gelatin methacryloyl (GelMA). NMR spectra were acquired at 30 ^o^C using a Bruker Avance III HD 400 MHz NMR spectrometer (Bruker BioSpin Corp., Billerica, USA), and the data were analysed using Bruker Topspin software 3.7.5. For NMR analysis, GelMA samples (10 mg/mL) and gelatin samples (4 mg/mL) were prepared in deuterium oxide (Sigma-Aldrich, Australia). The lysine peak (2.8–3.0 ppm) was used to confirm the methacrylation degree. The phenylalanine peak (7.1–7.4 ppm) was chosen to normalize the intensity of the lysine peak. The lysine peak shown in Fig. S1 confirmed a high methacrylation degree of 87 ± 5%, calculated according to the ratio of the relative peak integrations of the lysine protons in GelMA and gelatin.

### Platform and experiment setup

**Figure 1:**
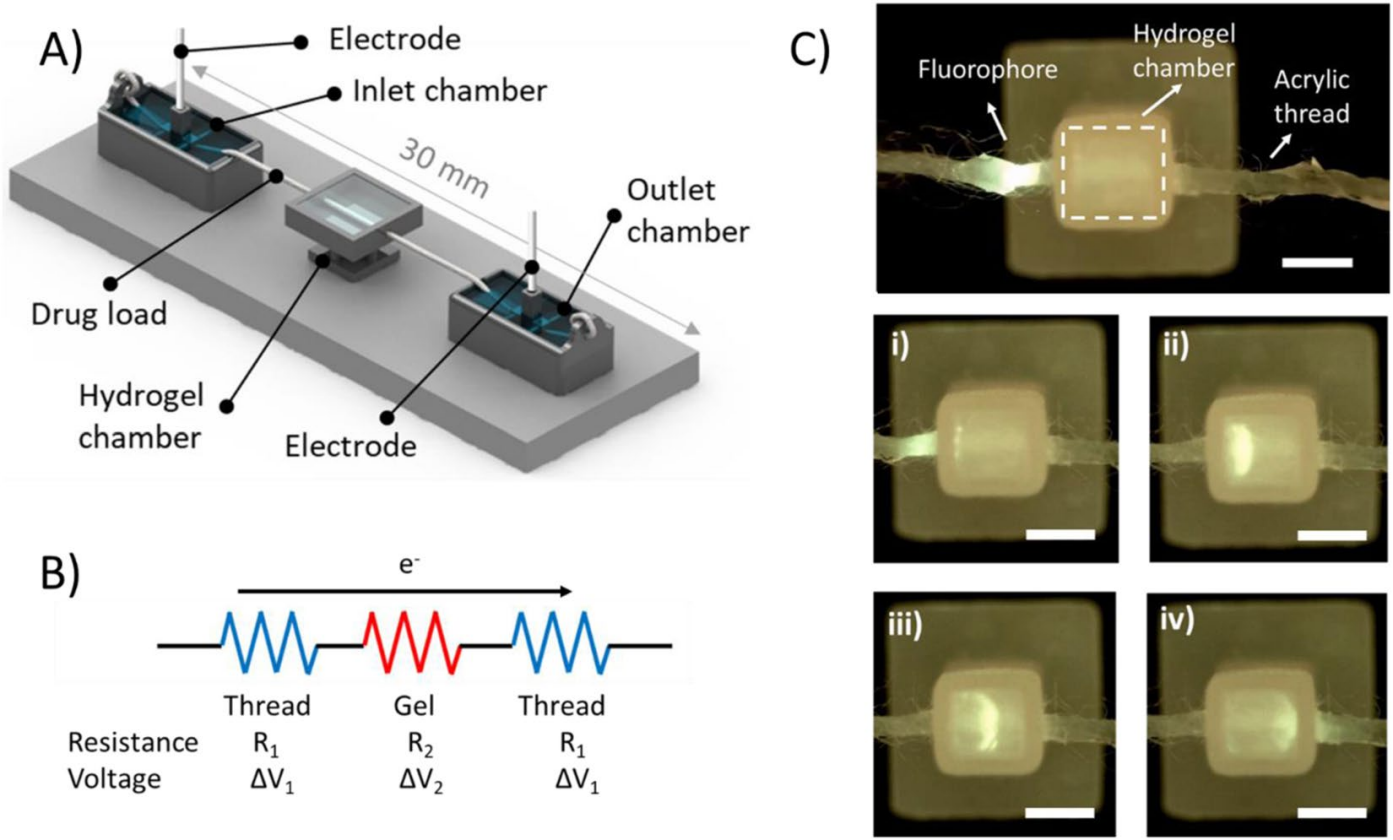
Schematic of the suture-GelMA hydrogel delivery platform (**A**) consisting of two buffer chambers with hydrogel chamber sitting in the middle, and a suture/thread wetted with preferred buffer to connect both liquid and hydrogel. Fluorophore (fluorescein) was loaded at 5 mm before the hydrogel chamber. Electric field was applied between inlet and outlet chambers. (**B**) A simple circuit diagram representing the contribution of each electrical component of the platform when electric field is applied. (**C**) Sequence of images depicting the parts and delivery process of fluorescein (a model compound) in GelMA at (i) 0.00, (ii) 2.30, (iii) 4.30 and (iv) 7.40 min. Images were picked from the top of the platform at 510 nm emission filter. Conditions: 5% GelMA and 45 s cross-linking (stiffness of 4.3 kPa), sample loading: 2 μL fluorescein drop at 1.0 μg/mL, current of 100 μA. 5 mM Tris/HEPES buffer was used for both inlet/outlet chambers and GelMA. Scale bar is 2 mm. Data presented here are based on 6 replicates.

As illustrated in Fig. 1A, experiments were conducted in a suture-GelMA hydrogel-based device. The platform consisted of two 3D printed inlet and outlet chamber at each end filled with 500 μL of 5 mM Tris/HEPES, a 3D printed hydrogel chamber sitting in the middle with 15 μL of 5% GelMA cross-linked for 45 s, and a 3D printed base to allow good alignment and support of these features. GelMA Inlet and outlet chambers had a tubular guide to introduce the electrode, ring to tie in the thread, and a basin. The total length of the thread from the inlet to the outlet chamber was 30 mm, with 2.5 mm embedded into the hydrogel. The hydrogel chamber was 2.5 × 2.5 × 2.5 mm (xyz), had two holes in each side allowing the thread to go in and out of the hydrogel. Features were designed using SolidWorks CAD software (SolidWorks Corp., Dassault Systemes, France). Designs were 3D printed using an Eden 260VS (Stratasys, MN, USA) with VeroClear or VeroWhite build material, and SUP707 water soluble support. SUP707 support material was removed by agitation in 2% NaOH for 2 hours using a benchtop orbital shaker, followed by 4-6 hours in water. Finally, printed parts were rinsed with Milli-Q water. To interconnect the system, a plasma-treated acrylic or surgical suture was placed at the front part of the inlet chamber, along the hydrogel chamber up to the outlet chamber. Prior to analysis, all threads and sutures were plasma treated for 1 min at 18 W in 0.2 Torr using Harrick Plasma Cleaner PDC-32G (Harrick Plasma, Ithaca, NY) connected to a PlasmaFlo Gas Flow Mixer PDC-FMG. A power supply, HVS448 High Voltage Sequencer 3000D-LC (LabSmith, Livermore, CA, USA) in conjunction with a voltage modulator were used to generate an electric field between the electrodes immersed in the inlet and outlet reservoirs. The output of the sequencer (voltage, current and conductivity) was controlled and measured by a computer through the Sequence Software (Labsmith, Livermore, CA).

### Bioactive molecule delivery in suture-GelMA hydrogel

Fluorescein velocities were measured at 3 GelMA concentrations (2.5, 5.0, 7.5%) and 6 cross-linking time (0, 10, 20, 30, 45 and 60 seconds). Fluorescein was prepared in the same buffer solution as the GelMA hydrogel and reservoirs (5 mM Tris/HEPES). A drop (2 μL) of fluorescein solution was placed upon the acrylic thread (shown as ‘drug load’ in Fig. 1A). An electric field was applied at a constant current of 100 μA unless specified otherwise. 5% GelMA with 45 s cross-linking and the same electrofluidic conditions and buffer conditions were carried out for the flow delivery control. To test bioactive delivery, the same experiment was repeated using rhodamine B, riboflavin, FITC-BSA, FITC-dextran. Resistance comparation was carried out using 4 different buffers (5 mM Tris/HEPES, 50 mM Tris/HEPES, 2.5% PBS, and 5% PBS). Drug delivery of fluorescein in several 9 suture threads were measured at 250 µA of current, 5% PBS buffer and 5% GelMA with 45 s cross-linking. All experiments were done in triplicate. Temperature was monitored using an infrared camera (Ex series with MSX, FLIR Systems, VIC, Australia) and kept at 25 ± 1 °C. A USB microscope AM4113T-GFBW (Dino-Lite Premier, Clarkson, WA, Australia) fitted with a blue light-emitting diode for excitation and a 510 nm emission filter was used to take fluorescence images and videos (resolution of 1.3 M pixels and 30fps). ImageJ (National Institutes of Health, http://rsb.info.nih.gov/ij/) was used to analyse the region of interest (ROI) and then monitor the mean fluorescence intensity value of the ROI versus time. The data was acquired from the moment the fluorescent compound band entered the hydrogel. This software was also used to determine the band position at each time, and velocity was calculated using division of band displacement by time. In all cases, position was referenced to the front part of the band.

A similar experiment was performed to study the delivery of dexamethasone 21-phosphate disodium salt (DSP) using 5% GelMA with 45 s cross-linking and 5% PBS as buffer. A drop (2 μL) of DSP (1.0 mg/mL) was placed on medical surgicryl PGA suture, and an electric field was applied at a constant current of 250 µA. Buffered solutions from the inlet and outlet chambers were collected from three experiments performed at two different running times (1 and 2 hours). Triplicates of the same experiment without electric field were carried out for the blank. Then the buffer solution from the chambers were collected. DSP content in all samples collected were measured by high pressure liquid chromatography (HPLC).

### HPLC analysis of drug-released samples

An Agilent 1260 Infinity HPLC system equipped with an Atlantis T3 5 µm 4.6 × 250 mm C18 Column (Waters, Australia) was employed for the study. A gradient HPLC method was used for the analysis of DSP using a UV detector at 242 nm. The mobile phase comprised A (20 mM ammonium acetate) and B (acetonitrile) with a flow rate of 1 mL/min. The initial mobile phase was maintained at 23% B for 5 min, and then changed linearly to 100% B in 1 min (5–6 min), maintained at 100% B for 4 min (6–10 min), and finally, reduced linearly to 23% B in 2 min (10–12 min) and maintained at 23% B for 4 min (12–16 min). The injection volume was 10 µL, and each sample was filtered through a 0.22 μm regenerated cellulose syringe filter (Phenomenex Australia Pty Ltd). DSP concentration within the chambers were determined using the parameters obtained from the calibration curve (70–1500 ppb).

### Statistical analysis

Statistical analysis was performed using the OriginPro 2015 statistical software academic version and Microsoft Excel statistical analysis. Data are presented as mean ± standard error of the mean. The value of n denotes the number of performed samples or the number of independently performed attempts. Analysis and comparisons between conditions and controls were analyzed by using one-way ANOVA to test for differences among five groups (unless specified otherwise) followed by Bonferroni’s post-hoc test.

## Results and Discussion

### Mechanical properties of soft biological tissues

**Figure 2:**
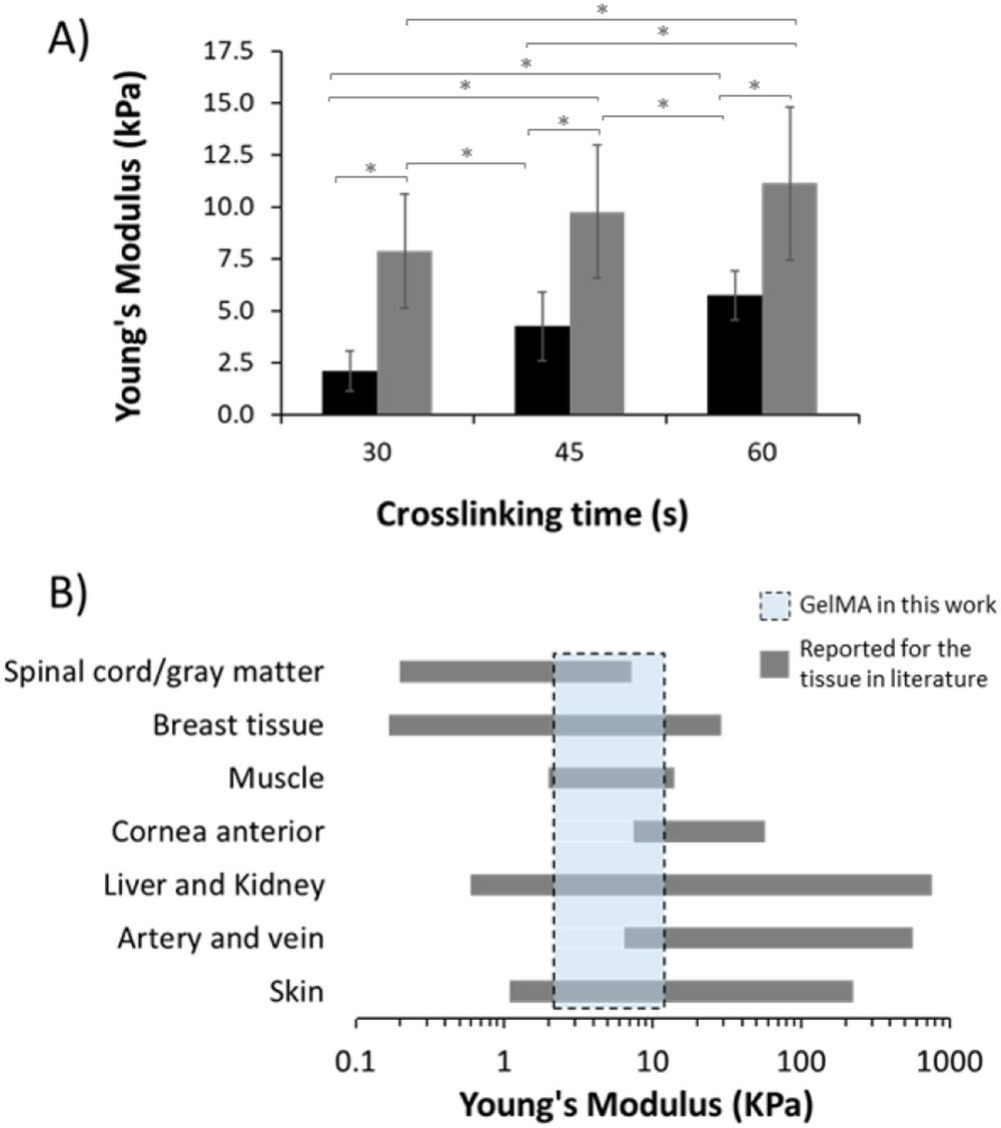
Tailoring mechanical properties of gelatin methacryloyl (GelMA) hydrogels to match soft tissues. (**A**) Young’s modulus for the hydrogels prepared from 5.0 (black) and 7.5% GelMA (grey). Error bars are based on the standard deviation of 5 replicates. *Indicates significant difference (P < 0.05). (**B**) Comparison of Young’s modulus developed in this work (dashed box) with the soft biological tissues (grey bars). Dashed box represents the region for 5.0-7.5% GelMA. The Young’s modulus values obtained for the GelMA hydrogels were compared with the values reported within the literature for 7 soft biological tissues^34,35^.

The mechanical properties of the GelMA hydrogel affect the diffusion and delivery of bioactive species^32^. GelMA is often described as mechanically tunable. Changes in photo-crosslinking conditions cause different rates and extent of reaction, thus altering the mechanical properties of GelMA^33–35^. In this work, the Young’s modulus was determined for 2 GelMA concentrations. Figure 2A shows a Young’s modulus of 2.1 ± 1.0, 4.3 ± 1.7, 5.7 ± 1.2 kPa for the 5.0% GelMA hydrogel photo-crosslinked for 30, 45 and 60 s, respectively, and 7.9 ± 2.7, 9.8 ± 3.2, 11.1 ± 3.7 kPa for the 7.5% GelMA hydrogel photo-crosslinked for 30, 45 and 60 s, respectively. As seen in Fig. 2B, values obtained matched those of several soft tissues, particularly normal skin, liver and kidney. The use of lower concentration GelMA (5%) provides gels that more closely mimic softer tissues such as spinal cord, gray matter or muscle, with the highest concentration gels (7.5%) being closer to cornea anterior, artery and vein.

### System and electrodynamic considerations

As illustrated in Fig. 1, the 3D printed platform enabling this study comprised of several parts: two reservoirs containing buffer and electrodes into which each end of the thread is immersed. The thread travels from one reservoir to the other through the gel. When current is applied between the two buffer chambers, a heterogeneous electrical system is created, as shown in Fig. 1B. Neither sutures/threads nor hydrogels are electrically conductive. Electrolyte serve as ionic conducting medium. When external electric field was applied, the electrokinetic phenomenon of electrophoresis was observed and ultimately caused the presence of a charged interface on the surface and the consequent migration of molecules. Electrofluidic dynamics depend mostly on the physicochemical properties of the thread, the physical properties of the hydrogel, and the composition of the electrolyte/buffer used^9,12^. Thread resistance (referred to the buffer suspended in the thread) changes with the thread diameter, length and wettability (material hydrophilicity), whereas hydrogel electrical resistance depends on gel dimensions, structure of crosslinked networks and water content (Fig. S2)^9^. Electrolyte composition is also important, as increases in concentration or conductivity will lead to higher currents. The magnitude of the applied current and polarity determine both speed and direction (migration) of the solutes upon the thread. If a bioactive species is present, its velocity would increase with the magnitude of the current^9^. Due to the change in cross-sectional area, the magnitude of the current density is higher along the bare thread, whereas it is substantially lower and spatially distributed within the hydrogel. Consequently, the bioactive velocity is reduced when it enters the hydrogel.

### Biomolecule delivery in GelMA hydrogels

#### Transport process

To achieve an effective flow and controlled biomolecule migration within hydrogels, plasma-treated (hydrophilic) acrylic thread (diameter to 670 µm) were used. As described in the Theory section available within the supplementary information, biomolecule transport is directly proportional to the voltage. Nevertheless, the rise in voltage must be compensated with a reduction in current, otherwise the power (product of voltage and current) can generate Joule heating. Therefore, in order to reduce the generated current and minimize Joule heating effects, a low conductivity buffer (5 mM Tris/HEPES, 62.72 Ω m, pH 7.8, ionic strength 3.38 mM) was used. The temperature in each set-up was monitored using an infrared camera, and was shown to be 25 ± 1 °C. This suggests the absence of any significant Joule effects during the course of delivery. Figure 1C shows a schematic representation of the hydrogel chamber before solute delivery (in this example fluorescein (FL), a negatively charged fluorophore), with FL shown dropped directly onto the inlet acrylic thread. As soon as a current of 100 μA was applied, the FL band migrated towards the anode (+) along the thread. When the band entered the hydrogel the velocity decreased as a result of the increased viscosity and the dissipation of the field within the gel. FL then spreads radially from the thread, to form a semi-sphere. Finally, as the band exits the gel the FL band becomes refocused onto the outlet thread. This can be viewed as a Movie in the Supplementary Information, which shows the delivery process. Less than 8 minutes was needed to transfer the FL band along the thread through the hydrogel, and then exit on the thread. FL travelled through the hydrogel at a constant velocity of 7.17 ± 0.50 μm/s (n = 6).

#### Delivery of bioactive species

**Figure 3:**
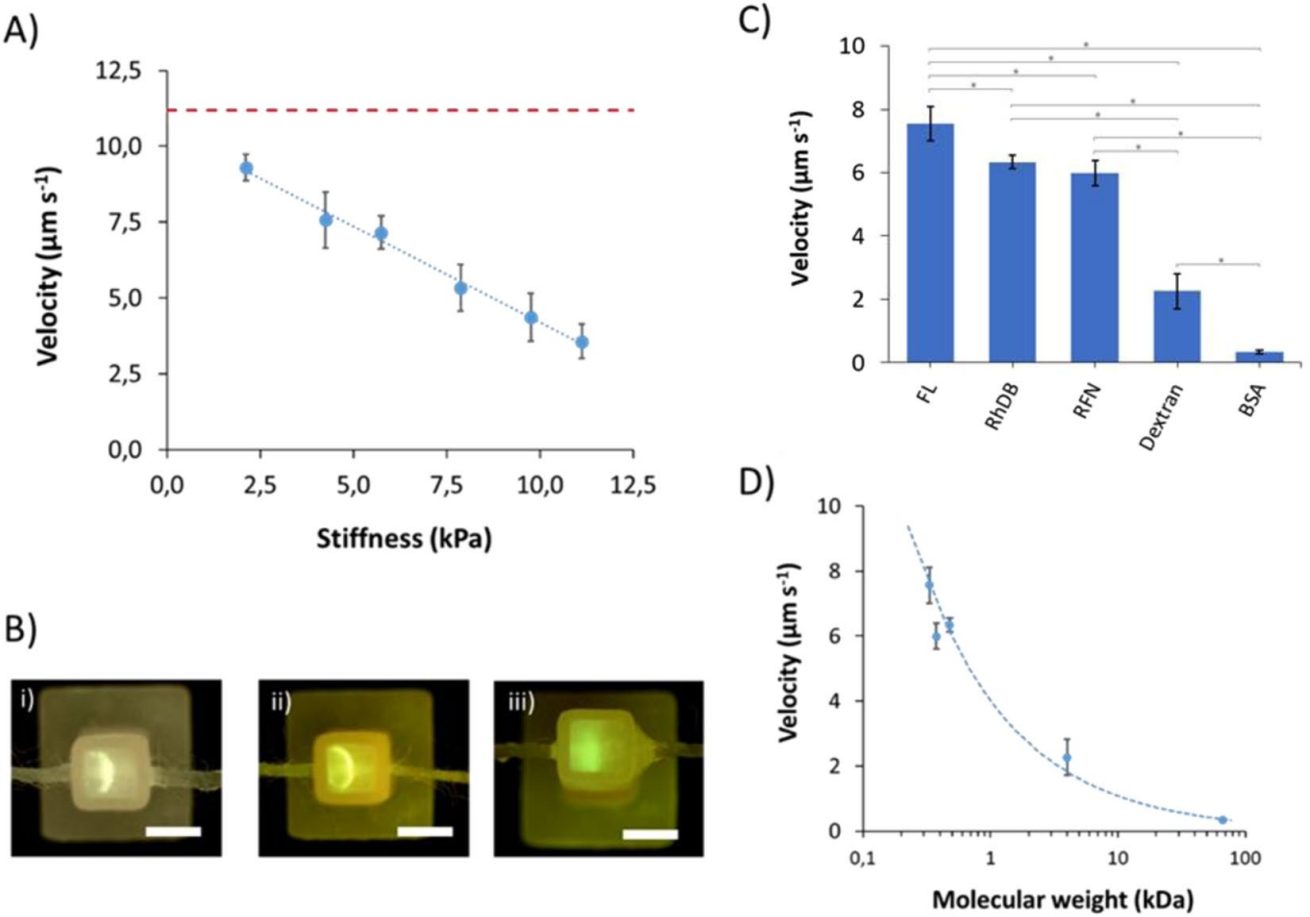
Delivery in soft tissue models based on GelMA hydrogels. In all cases 0.5 pg of compound was dropped on acrylic thread and 100 μA was applied for electric field (unless specified otherwise). 5 mM Tris/HEPES buffer was used for both inlet/outlet chambers and GelMA. Error bars are based on the standard deviation of 3 replicates. (**A**) Speed of fluorescein (FL, a model compound) as a function of the stiffness (Young’s modulus). Trend line was y = −0.63 × +10.52, R-squared = 0.9912. Red dashed line represents the velocity at 0 crosslinking (11.2 ± 0.4 μm/s). (**B**) Representative pictures were taken for (i) 5% GelMA (45 s cross-link, stiffness of 4.3 kPa) at 100 μA, (ii) 7.5% GelMA (30 s cross-link, 7.9 kPa) at 100 μA, and (iii) 7.5% GelMA (60 s cross-link, 11.1 kPa) at 150 μA. Scale bar is 2.5 mm (**C**) Velocity of 5 different compounds and bioactive species: fluorescein (FL), rhodamine B (RhDB), riboflavin (RFN), BSA, dextran) in a GelMA hydrogel with a stiffness of 4.3 kPa, conditions were the same as Fig. 1. *Indicates significant different (P < 0.05). (**D**) Velocity as a function of the molecular weight of the compound. Speed in hydrogel declines with molecular weight following a trend of y = 4.02 × −0.57, R-squared = 0.9879.

A range of exposure times and GelMA concentrations were tested in order to evaluate delivery with respect to gel stiffness. Shown in Fig. S3 are the data for FL velocity within 2.5, 5.0, and 7.5% GelMA, as a function of crosslinking time from 0, 10, 20, 30, 45 and 60 seconds. The velocities for GelMA 5% and 7.5% were compared with the respective Young’s moduli and are shown in Fig. 3A. To compare results, all experiments were carried out using a 100 μA current. p < 0.05 was considered to be statistically significant. The velocity decreased linearly with increasing the hydrogel stiffness as characterized by respective Young’s modulus. A velocity of 9.3 ± 0.4 μm/s at a Young’s modulus of 2.1 kPa was found, compared with 11.2 ± 0.4 μm/s at zero crosslinking. In contrast, for a tougher hydrogel with Young’s modulus of 11.1 kPa, velocity was much slower (3.6 ± 0.6 μm/s). Increasing the concentration of a macromer, as is the case with GelMA, results in formation of more densely crosslinked polymer network that is responsible for an increase in stiffness and therefore a reduction in ion mobility^25,27^ – similar to the influence of agarose concentration in gel electrophoresis, commonly known as sieving matrix. A resultant reduction in the ion mobility produced sharper solute bands within the gel (Fig. 3Bi-ii). An excessive increase of cross-linking caused an increase in stiffness within the hydrogel, hindering the delivery. This phenomenon was observed within hydrogels of stiffness >15 kPa.

Under the same conditions, a higher electric field provides faster solute delivery, up to a point, when if the electric field is too high, the infusion of biomolecules into the hydrogel cannot keep up with electrolyte movement and so accumulates at the thread-gel interface. This could cause a localized increase in ion concentration and therefore increase Joule heating and posterior hydrogel swelling. This was the case for a hydrogel with stiffness of 11.1 kPa at a current of 150 μA. Normal delivery was observed for the first two minutes, at which point the hydrogel began to swell, causing disruption of the band (Fig. 3Biii).

Figure 3C,D show the velocities of several bioactive molecules within the GelMA hydrogel, ranging from small metabolites to polysaccharides, to proteins, each with different charge and functionality. While migration was relatively rapid along the acrylic thread, each slowed rapidly as they entered the hydrogel. Small molecules such as FL, Rhodamine B (RhDB), and Riboflavin (RFN) migrated faster, with velocities of 7.57 ± 0.55, 6.34 ± 0.20, 5.99 ± 0.40 μm/s, respectively. Different migration directions were observed depending on the solute charge. For instance, FL (-) migrated to the anode, whereas the polarity needed to be switched for delivery of RhDB (+). Velocities for larger molecules such as dextran (4 kDa, 2.26 ± 0.55 μm/s) and BSA (66.5 kDa, 0.33 ± 0.07 μm/s) were substantially slower. This was caused by the hydrogel matrix, where smaller molecules moved and migrated more rapidly, as they could move freely through the polymer networks. Compared with small molecules, polysaccharides and proteins migrated slowly, being BSA the slowest. Statistical analyses were performed by one-way ANOVA. P < 0.05 was considered to be statistically significant, except between RhDB and RFN. This difference is insignificant due to their similarity in polarity and molecular weight.

#### Flow delivery control

**Figure 4:**
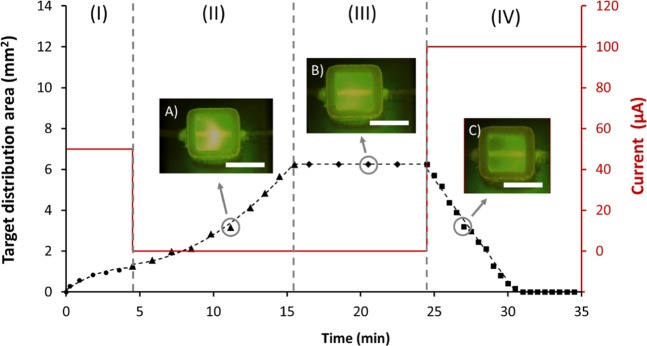
Controlled delivery via electric field into 3D GelMA hydrogel supports. Distribution area of fluorescein (FL, a model compound) within hydrogel with stiffness of 4.3 kPa as a function of time in 4 different steps. (I) band delivery, 50 μA, 4.5 min (•); (II) molecular diffusion, 11 min (▲); (III) equilibration, 9 min (♦); and (IV) excess removal, 100 μA, 10 min (■). Solid line represents the current applied. Representative images were taken at minutes 11.17 (insert **A**), 20.50 (insert **B**), and 27.00 (insert **C**). Trend line for excess removal was y = −0.9973 × +0.445, R-squared = 0.9887. Scale bars is 2.5 mm. Conditions were the same as for Fig. 1.

By controlling electric field and polarity, small molecules, drugs or growth factors can be dispatched in a controlled manner throughout the gel. The advantages of this approach are that the rate of delivery can be controlled and, by controlling field on/field off times, spatial control over the displacement of the molecule is achieved. Figure 4 shows a plot for 4-step controlled delivery of a solute band into the GelMA hydrogel: (I) band delivery from thread to the hydrogel (field on); (II) diffusion through the gel (field off); (III) equilibration (field off); and (IV) removal (field on). The spatial distribution of FL fluorescence was monitored versus time to determine the amount of FL in the hydrogel for each step of the process. This light was monitored using a microscope and images/videos were treated through ImageJ. Light threshold was kept constant and the FL distribution area was determined by selecting the ROI (region of interest) area. In this case, 0.5 pg of FL loaded upon the thread inlet segment was delivered as a band towards the hydrogel, when a current of 50 μA was applied. In step (I), the radial distribution increased as the band entered in the hydrogel at a constant velocity of 3.53 μm/s. A slight increase in area was observed due to band broadening of the solute zone. Once the band reached the middle of the gel, the voltage was turned off (at 4.5 min). Then, as shown in step (II), FL diffused into the 3D hydrogel space – as area per unit time (with an average of 0.45 mm^2^/min), described by Fick’s law. Approximately 11 min were needed to cover the entire 3D space of the hydrogel with FL. Although 100% of the area was occupied, a concentration gradient from the thread source to the gel walls was observed. Therefore, in step (III) and with voltage still off, the system was left for an extra 9 minutes to equilibrate and achieve a uniform concentration throughout the hydrogel. Line-scanning mode of ImageJ was used to determine the light intensity vs. single pixel-wide to recreate a plot of intensity profile along the delivery axis. Uniform concentration throughout the hydrogel was set when peak height was <10 a.u. (pixel intensity), less than 3 times the standard deviation of noise level. No loss of FL out of the hydrogel was observed during the 20 minutes without any applied electric field. Finally, in step (IV), the electric field was again activated at 100 µA to remove the FL. The removal rate was constant over time, taking 6.03 min to eliminate FL completely from the platform. Removal velocity of the FL was measured to be 6.90 μm/s (image C in Fig. 4). Note that at 100 μA the speed of the FL was almost doubled compared with step (I) where the current was only 50 μA.

### Drug delivery using sutures

**Figure 5:**
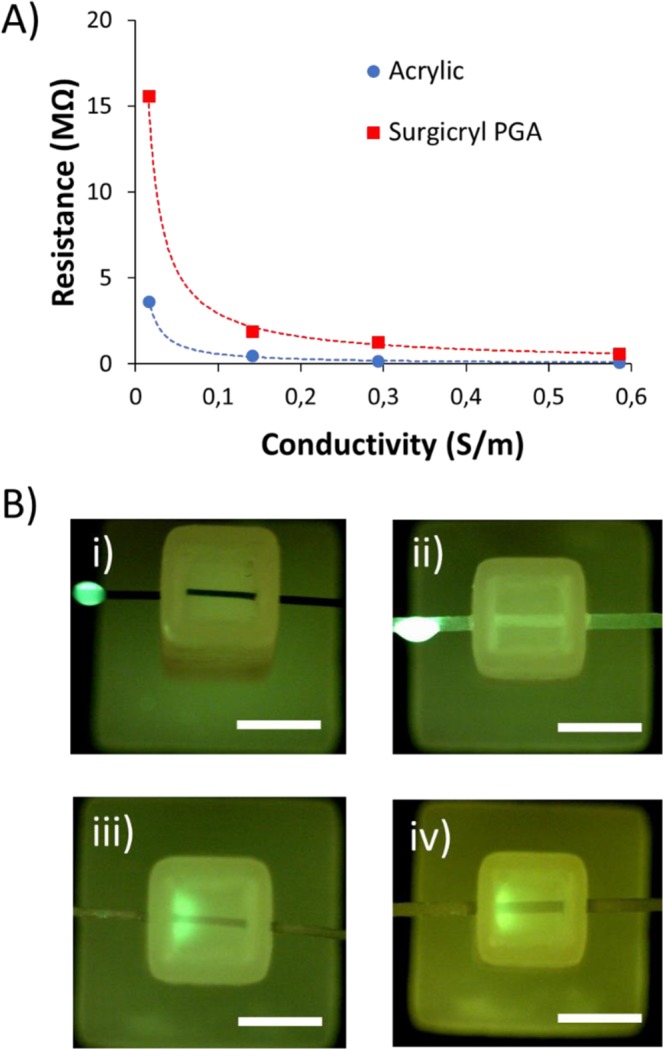
(**A**) Electrolyte resistance versus conductivity comparison between acrylic thread (diameter 670 µm) and surgicryl PGA (USP 0). Electrolytes were 5 mM Tris/HEPES (0.0159 S/m), 50 mM Tris/HEPES (0.141 S/m), 2.5% PBS (0.293 S/m), and 5% PBS (0.585 S/m) (**B**) Representative pictures depicting the parts and delivery process of fluorescein (FL, a model compound) taken after 4 minutes of applied current for 4 sutures: (i) Nylon (Ethilon, monofilament); (ii) Polyamide (Supramid, twisted and coated); (iii) Polyglactin 910 (Vycril, braided and coated); (iv) Polyglicolic acid (Surgicryl, braided and coated). Stiffness of the hydrogel was 4.3 kPa. Sample was loaded upon thread inlet segment with a 2 μL drop of a 1.0 μg/mL solution of FL. Scale bar is 2.5 mm.

For an effective targeted delivery, the drug should move quickly in the suture but slowed down when it is diffused off the suture to the gel, so that the desired biological effect can be produced. This would benefit several biological processes such as more efficient passive diffusion to a cell membrane or increase drug absorption for those with low permeable coefficients. To achieve that, modifications in electrolyte resistances were carried out, thus minimizing the electric field applied in the hydrogel and therefore the velocity (Equation 6S). The performance of a number of commercially available sutures was investigated using several electrolytes with increasing conductivity. Figure 5A shows the total resistance (R_T_) for the electrolytes within acrylic thread with polyglicolic acid (PGA) suture using 4 different biological buffers with conductivities ranging from 0.0159 to 0.585 S/m. The resistance is uniquely determined by the amount and conductivity of liquid that the thread is holding. Reducing the suture diameter (PGA with low USP size) and using a braided structure saw the total resistance being increased, allowing higher buffer concentrations to be used, typically more compatible for cell viability. Using 5% PBS (0.585 S/m) at a constant current of 250 μA, voltage was reduced to only 5.42 V between each extreme of a 15 mm^3^ hydrogel (2.5 mm long, 2.17 V/mm). This voltage was considered acceptable although it could be lowered further by increasing the length of the hydrogel chamber. Using the previous buffers (0.0159 S/m), a current of 250 μA would increase too much the voltage in the hydrogel, causing Joule heating effects and swelling.

Nine commercially available sutures produced from different materials, structures, sizes and water absorption characteristics were tested (Table 1). From these materials, polyester, polyglactin 910, glycolide/lactide copolymer, and polyglycolic acid created adequate fluid connection between the electrodes and the hydrogel (Fig. 5B). Initial delivery speeds ranged from 2.3 to 3.4 μm/s. However, the velocity decreased when the compound fully entered into the gel. As commented previously, using 5% PBS and 250 µA the voltage in the hydrogel was very low. For this reason, the measurement of the velocity in GelMA was extremely challenging and it is not shown. Structurally, braided sutures presented better connectivity, as they exhibit greater flow-through properties, arising from the open braided structure, thus allowing a continuous and homogeneous distribution of the electrolyte. However, braided silk sutures, a protein based natural suture from the species *Bombyx mori*, could not be used as these commercial sutures are typically processed to remove the natural waxes and gums, and coated with a special wax mixture that prevents electrolyte penetrating into the filaments^36^. Overall, the sutures which showed acceptable delivery performance can be applied in main medical indications (skin, gastro-intestinal, uterus, hernia, orthopedics, ophthalmic procedures, oral cavity, and muscle). Detailed information on indication and contra-indication from these sutures can be found in Table S1 from the supporting information.

**Table 1:** Chemical composition, structure, absorbability and size of sutures. Drug delivery speed of FL was measured for first 5 minutes of delivery at 250 μA, 5% PBS. Conditions were the same as for Fig. 5. Standard deviation based on 3 experiments.

Name	Composition	Properties	Absorbable	Size (USP)	Delivery (μm/s)
Monocryl	Poliglecaprone 25	Monofilament	91 - 119 days	3/0	No
Prolene	Polypropylene	Monofilament	No	3/0	No
Ethilon	Nylon	Monofilament	No	3/0	No
Supramid	Polyamide based	Twisted and coated	No	0	No
Silk	Silk	Braided	No	3/0	No
Polyester	Polyester	Braided	No	4/0	3.4 ± 0.4
Vicryl	Polyglactin 910	Braided and coated	56 - 70 days	3/0	3.0 ± 0.3
Polysorb	Glycolide/Lactide copolymer	Braided and coated	56 - 70 days	3/0	2.9 ± 0.3
Surgicryl	Polyglycolic acid	Braided and coated	60 - 90 days	0	2.3 ± 0.3

To demonstrate a potential application for controlled localised delivery, the delivery of an anti-inflammatory drug, dexamethasone 21-phosphate disodium salt (DSP), was investigated. DSP is a prodrug of dexmathasone that is used to treat many different inflammatory conditions including skin inflammatory diseases and wound treatment. To monitor the amount of DSP delivered, buffer electrolyte from both inlet and outlet was examined using an HPLC. With an applied current of 250 μA, DSP was moving slowly enough to be diffused off the suture and spread within the 3D hydrogel space. After applying the electric field for 1 hour, DSP was only observed in the outlet reservoir at very low concentrations (0.096 ppm), indicating 97.6% was still in the hydrogel. When the electric field was applied for 2 hours, the percentage remaining within the hydrogel was reduced to 81.1% of the drug. Furthermore, no DSP was found in the inlet reservoir, proving that 100% of DSP was directed to the desired location and remained there for this period; which would be essential for its actuation in the target area. The applied electric field may also cause compounds presenting within the hydrogel to migrate toward their oppositely charged electrode. Thus, there is significant potential here to simultaneously monitor metabolites or products arising from the delivery of a bioactive agent. Moreover, this setup could also be used to study drug-cell interactions since metabolites and biomolecules of potential interest can be removed at will from the hydrogel for their analytical determination, leaving the surviving cells in place.

## Conclusion

We have developed a system to release bioactive molecules such as small molecules, drugs, polysaccharides, or proteins in a controlled manner via an electric field into tissue-like 3D hydrogel supports. Threads and commonly employed sutures were successfully assembled in GelMA-based hydrogels to facilitate the introduction of bioactive species, and to deliver them via controlled diffusion to fill a desired 3D space. The advantages afforded by this delivery system are: (1) delivery can be turned on and off when needed; (2) during delivery the rate can be controlled; (3) by alternating the field on/off, spatial control over the drug delivery is possible; (4) the thread/suture chemistry can be modified to afford control over the interaction and hence release of the bioactive species.

Targeted drug delivery currently covers all means of delivering a bioactive species to a target region, point, or receptor within a biological system; and covers bulk targeted delivery (to a region) and selective drug delivery (to a specific receptor point, cell type or other target biomolecule/species). Surgical sutures and electric field were put together to deliver bioactive species into tissue-like materials. This work could use commonly employed sutures as the delivery conduit to deliver anti-inflammatory drugs for treatment of skin inflammatory diseases and wound infection. In addition, these early assemblies demonstrate the possibility of using such scaffolds for applications in bio-sensing and drug screening, and add to the exciting and rapidly growing field of fibre-based diagnostic and delivery technologies^37^.

## Supplementary information

Supplementary Information.

Supplementary Movie 1.
