# Supplementary material for: Electrofluidic control of bioactive molecule delivery into soft tissue models based on gelatin methacryloyl hydrogels using threads and surgical sutures
Source: Sci Rep. 2020 Apr 28;10:7120. doi: 10.1038/s41598-020-63785-z (PMC7188853; doi:10.1038/s41598-020-63785-z)
Supplement: Supplementary file 1 — Supplementary Information. [file 41598_2020_63785_MOESM1_ESM.docx]

Supporting Information

Electrofluidic control of bioactive molecule delivery into soft tissue models based on gelatin methacryloyl hydrogels using threads and surgical sutures

*Joan M. Cabot†, Luciana Y. Daikuara†, Zhilian Yue, Patricia Hayes, Xiao Liu, Gordon G. Wallace*, Brett Paull**

*Email: [brett.paull@utas.edu.au](mailto:brett.paull@utas.edu.au)

*Email: [gwallace@uow.edu.au](mailto:gwallace@uow.edu.au)

This document contains the following supplementary figures and information further describing our study:

- Theory 2
- Movie1: Delivery in GelMA 3
- Figure S1: NMR spectra 3
- Figure S2: Schematic diagram 4
- Figure S3: Speed of FL GelMA 4
- Table S1. Compilation of indication/contra-indication 5

**Theory:**

Electric field intensity, E (V/m), in a heterogenous system formed by a connection of different cross-sectional parts (2 equal length thread/surgical suture and 1 hydrogel) is described by the following relationship:

$$E=\frac{V}{L}=\frac{V_{T}}{L_{thd}+\frac{L_{Gel}}{\chi^{2}}}=\frac{V_{T}}{L_{thd}+\frac{L_{Gel}}{\left( \frac{A_{Gel}}{{\pi r}^{2}} \right)^{2}}} (1S)$$

where $V_{T}$ is the voltage applied (V), $L_{thd}$ and $L_{Gel}$ are the length of the individual parts of the thread and GelMA hydrogel (m), $\chi$ is the ratio of the internal cross-sections of the parts, $A_{Gel}$ is the cross-section area of the hydrogel (m^2^), and r the radius of the thread (m). Therefore, voltage in GelMA hydrogel ($V_{Gel}$) and thread ($V_{thd}$) can be calculated by:

$$V_{thd}=IR_{thd}=I\frac{L_{thd}\rho_{thd}}{\pi r^{2}}=EL_{thread} (2S)$$

$$V_{thd}=IR_{Gel}=I\frac{L_{Gel}\rho_{Gel}}{A_{Gel}}=EL_{Gel} (3S)$$

where I is the current intensity (A), $R_{thd}$ is the resistance of the thread (Ω), $R_{Gel}$ the resistance of the gel (Ω), and $\rho_{thd}$ and $\rho_{Gel}$ are the respective resistivities of the thread and the gel (Ω cm). Knowing I, voltage, and dimension of the thread and gel, $\rho_{thd}$ and $\rho_{Gel}$ can be calculated using a modified equation from 2S and 3S.

The change in resistance (f) can be calculated by:

$$f= \frac{R_{thd}}{R_{Gel}}=\frac{\frac{L_{thd}\rho_{thd}}{\pi r^{2}}}{\frac{L_{Gel}\rho_{Gel}}{A_{Gel}}} (4S)$$

Supposing that$\rho_{thd}=\rho_{Gel}$, when a hydrogel of 2.5×2.5×2.4 mm (xyz; 15 μL) and a thread of radius 335 µm are used, the resistance within the hydrogel is 17-times lower. Whereas, if the thread radius is reduced to 100 µm, hydrogel resistance becomes 190-times lower.

Velocity of the band ($\bar{v}$, m/s) can be calculated as a function of the effective length ($L_{ef}$, m) and time, or the apparent mobility ($\bar{\mu}$, m^2^V^-1^s^-1^), from the following:

$$\bar{v}=\frac{L_{ef}}{t}=\bar{\mu}E (5S)$$

Since the flow of the bulk solution is 0, $\bar{\mu}$ equals the electrophoretic mobility ($\mu_{e}$) and therefore this equation can be rewritten as:

$$\bar{v}=\mu_{e}E=\frac{qE}{6\pi\eta r}=\frac{qV}{6\pi\eta r{(L}_{thd}+L_{Gel})} (6S)$$

where q and r are the charge of the respective ion charge and radius of the molecule, and $\eta$ the viscosity (Pa·s) of the solution.

**Movie1:** Delivery process of fluorescein in GelMA at Video recorded from top at 510 nm emission filter. Conditions: 5% GelMA, 0.06% v/v LAP, 60 s crosslinked, sample loading: 2 μL drop at 1.0 μg/mL, current of 100 μA. Speed increase: 16-times.


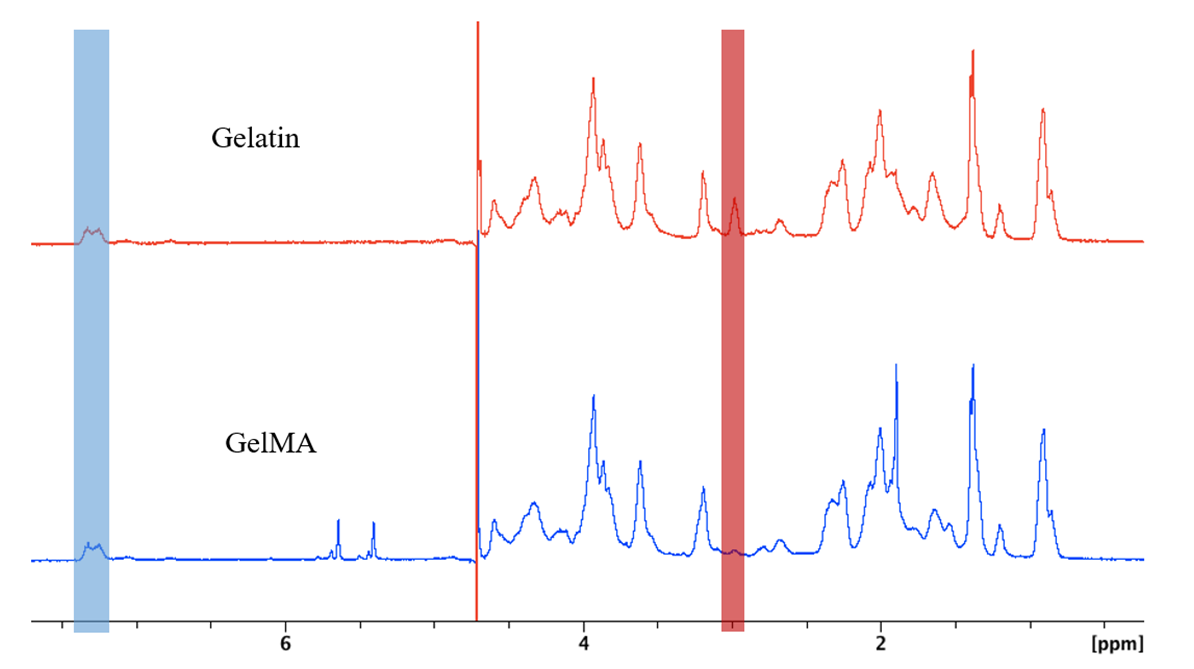


**Figure S1.** NMR spectra of gelatin (red) and GelMA (blue). The red band is the lysine peak (2.8–3.0 ppm) used to quantify the degree of methacrylation. In each spectrum, the integration of the lysine peak is normalised by that of the phenylalanine peak (7.1–7.4 ppm, blue band).


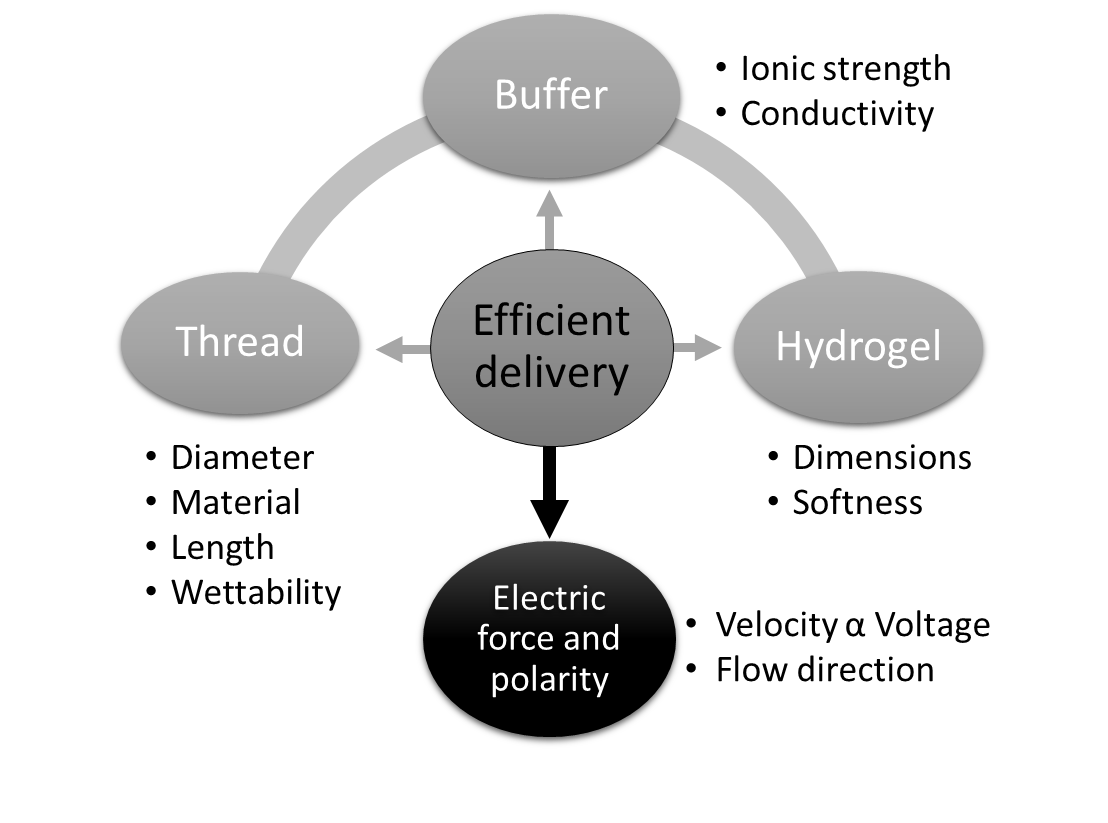


**Figure S2**. Schematic diagram depicting each of the electrodynamic considerations for the thread – hydrogel system.


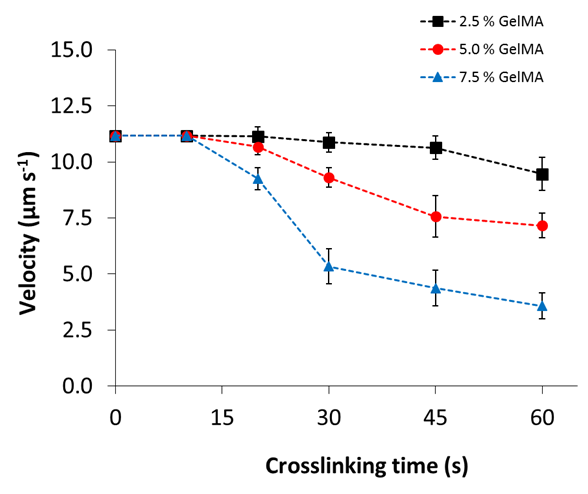


**Figure S3:** Speed of FL in the hydrogel prepared from 2.5, 5.0, or 7.5 % of GelMA as a function of the crosslinking time. The velocity clearly decreased with increasing GelMA concentration and photo-crosslinking time. At low cross-linking times (≤~20 s), where the hydrogels were softer, velocities were much faster, close to that prior to the photocrosslinking (velocity of 11.17 μm/s). Conditions: 0.5 pg of sample was dropped on acrylic and 100 μA was applied for electric field. Error bars are based on the standard deviation of 3 replicates.

Table S1. Compilation of indication/contra-indication from sutures studied in this work.

| **Name** | **Composition** | **Brand** | **Indication** | **Contra-indication** |
| --- | --- | --- | --- | --- |
| **Monocryl** | Poliglecaprone 25 | Ethicon | General soft tissue approximation and/or ligation; subcuticular skin closure | Cardiovascular or neurological tissues, microsurgery or ophthalmic surgery; where extended approximation of tissue under stress is required. |
| **Prolene** | Polypropylene | Ethicon | General soft tissue approximation and/or ligation; cardiovascular, ophthalmic, and neurological procedures. | Where extended approximation of tissue is required. |
| **Ethilon** | Nylon | Ethicon | General soft tissue approximation and/or ligation; cardiovascular, ophthalmic, and neurological procedures. | Where permanent retention of tensile strength is required. |
| **Supramid** | Polyamide | SMI | General soft tissue approximation and/or ligation; skin closure. | Where extended approximation of tissue is required . |
| **Silk** | Silk | Ethicon | General soft tissue approximation and/or ligation; cardiovascular, ophthalmic, and neurological procedures. | Where permanent retention of tensile strength is required. |
| **Vicryl** | Polyglactin 910 | Ethicon | General soft tissue approximation and/or ligation; ophthalmic procedures. | Cardiovascular and neurological tissues; where extended approximation of tissue is required. |
| **Polysorb** | Glycolide/lactide copolymer | Covidien | General soft tissue approximation and/or ligation; ophthalmic procedures. | Cardiovascular and neurological tissues; where extended approximation of tissue is required. |
| **Surgicryl** | Polyglycolic acid | SMI | General soft tissue approximation and/or ligation; general surgery, skin closure, gastrointestinal surgery, gynaecology, obstetrics, plastic surgery, urology, ophthalmic surgery, orthopaedics. | Cardiovascular and neurological tissues; where extended approximation of tissue is required. |
| **Polyester** | Polyester | Keebomed | General soft tissue approximation and/or ligation; cardiovascular, ophthalmic, and neurological procedures. | Only veterinary use in the USA / can be used in human or veterinary procedures in other countries. |
